# Glypican-5 suppresses Epithelial-Mesenchymal Transition of the lung adenocarcinoma by competitively binding to Wnt3a

**DOI:** 10.18632/oncotarget.12945

**Published:** 2016-10-27

**Authors:** Siwei Wang, Mantang Qiu, Wenjia Xia, Youtao Xu, Qixing Mao, Jie Wang, Gaochao Dong, Lin Xu, Xin Yang, Rong Yin

**Affiliations:** ^1^ Department of Thoracic Surgery, Nanjing Medical University Affiliated Cancer Hospital, Jiangsu Key Laboratory of Molecular and Translational Cancer Research, Jiangsu Biobank of Clinical Resources, Cancer Institute of Jiangsu Province, Nanjing 210009, China; ^2^ The Fourth Clinical College of Nanjing Medical University, Nanjing, 210000, China; ^3^ Department of Oncology, The Third Hospital of Soochow University, Changzhou, 213003, China

**Keywords:** Glypican-5 (GPC5), lung adenocarcinoma (LAC), Epithelial-Mesenchymal Transition (EMT), Wnt/β-catenin signaling pathway

## Abstract

We previously demonstrated that Glypican-5 (GPC5), one of the members of heparan sulfate proteoglycan, was a novel tumor metastasis suppressor in lung adenocarcinoma (LAC). However, it remains unclear how GPC5 suppresses lung cancer metastasis. Here, we found over-expression GPC5 induced significant Epithelial-Mesenchymal Transition (EMT) process of A549 cells *in vitro*. Bioinformatic analysis of RNA sequencing data indicated that GPC5 was co-expressed with EMT related markers, E-cadherin and Vimentin. Wnt/β-catenin signaling pathway was also significantly enriched after overexpressing GPC5. Further *in vitro* experiments demonstrated that overexpressing GPC5 could block the translocation of β-catenin from cytoplasm to nucleus and therefore inactivate the Wnt/β-catenin signaling pathway by competitively binding to Wnt3a. Subsequent rescue experiments demonstrated that GPC5-induced metastatic phenotype and EMT process suppression were significantly reversed when cells cultured in Wnt3a conditioned media. By establishing the metastatic model in severe combined immune deficiency (SCID) mice, we also demonstrated that overexpressing GPC5 suppressed LAC migration and accordingly alerted EMT related markers, which including up-regulated E-cadherin and down-regulated Vimentin in both lung and liver metastasis. Finally, clinical samples of LAC further validated that GPC5 expression was positively correlated with E-cadherin, and negatively correlated with both Twist1 and MMP2. Taken together, these data suggested that GPC5 is able to suppress the LAC metastasis by competitively binding to Wnt3a and inactivating the Wnt/β-catenin signaling pathway. Our findings expanded the role and the molecular mechanism of GPC5 on malignant bionomics of LAC.

## INTRODUCTION

In recent years, lung cancer rates and deaths are increasing dramatically around the world. According to the reports in 2014, the overall rate of incidence of lung cancer is still high and lung cancer is the most common cause of cancer death [1]. Lung adenocarcinoma (lung adenocarcinoma, LAC) and squamous cell carcinoma (squamous cell carcinoma, SCC) are the two main pathological types of non-small cell lung cancer (NSCLC). According to the world health organization (WHO), due to increasing national tobacco control and variation of the environment pollutants, the incidence of lung cancer of different pathological types is quietly changing. The incidence of LAC (31.5%) has gradually exceeded SCC (29.4%), and it presents rapid growth trend [2]. The trend is more obvious in China (LAC vs. SCC: 52% vs. 33%) [3].

Glypican-5(GPC5), a membrane-associated heparan sulfate proteoglycan (HSPG), plays a crucial role in cell proliferation and metastasis in breast cancer [4], lymphoma [5] and rhabdosarcoma [6]. Among these studies, molecular mechanisms associated with chromosomal amplification and Hedgehog signaling had been demonstrated. In addition, a multi-institutional genome-wide association study identified nucleotide polymorphism in the GPC5 gene associated with susceptibility to lung cancer in never smokers [7]. Upregulation of GPC5 in non-small cell lung cancer was also found promoting cancer cell migration [8]. In our previous study [9], we first reported GPC5 expression was significantly lower in the tumors of patients with lymph nodes metastasis (Stage N1-2) than in the tumors of patients without lymph nodes metastasis (Stage N0). Overexpressing GPC5 in NSCLC cell lines significantly inhibited their migration, invasion, and proliferation activities and also induced G1/S phase arrest of the cells *in vitro*. We suggested that GPC5 might be a novel metastasis suppressor gene in NSCLC. However, it remains unclear how GPC5 suppresses lung cancer metastasis.

Since Epithelial-Mesenchymal Transition (EMT) process has been proven to be an essential step of lung cancer metastasis [10]. Therefore, in the present study, we mainly focused on whether overexpressing GPC5 could alter EMT phenotype *in vitro* and *in vivo* at first. Subsequently we performed RNA sequencing and bioinformatic analysis for potential mechanisms screening. Further *in vitro* luciferase reporter assay, immunoprecipitation, as well as rescue experiments were performed to confirm how GPC5 suppresses the EMT process of LAC. And finally the correlation between the expression of GPC5 and EMT related genes were validated in clinical LAC specimens.

## RESULTS

### GPC5 mRNA expression is correlated with lung adenocarcinoma (LAC) lymphatic metastasis

**Figure 1:**
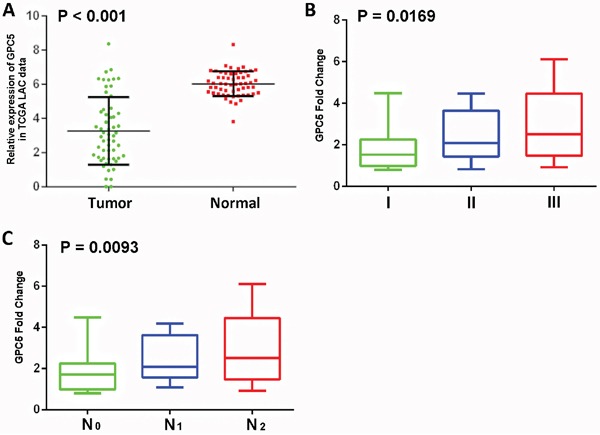
Expression levels of GPC5 in lung adenocarcinoma (LAC) and its clinical significance **A.** GPC5 mRNA expression in the TCGA LAC RNAseq dataset (normal n=57 vs tumor n=57, Fisher's t-test, *p* < 0.001). **B** and **C.** GPC5 down-regulated is associated with greater pTNM stage (p = 0.019) and N stage (*p* = 0.0093) (normal vs. tumor).

To investigate the expression of GPC5 in human LAC, we extracted the data of 57 paired LAC samples from TCGA datasets and 134 paired LAC samples from the previous literature, respectively [9]. The analysis confirmed that the expression of GPC5 gene was lower in lung cancer tissues compared with adjacent noncancerous tissues (Figure 1A). According to the Figure 1B and Supplementary Table S1, there was significantly difference in the GPC5 expression among groups classified by pTNM stage (*p* < 0.001; *p*=0.015, respectively). Notably, GPC5 expression in the stage N1-2 group in Supplementary Table S1 was remarkably lower than that in the stage N0 group (*tumor vs. normal*), and the data coincided with the TCGA results (*normal vs. tumor*) (Figure 1C). In addition, lower GPC5 expression was also correlated with poorer pathologic differentiation (Supplementary Table S1; *p*= 0.001). As shown in Supplementary Table S2, spearman correlation analysis indicated that the decreased expression of GPC5 mRNA in the tumor was positively correlated with N stage, pTNM stage and differentiation. All these outcomes indicated that the GPC5 expression level is correlated with the lymphatic metastasis ability of LAC cells.

### Overexpressing GPC5 inhibits the Epithelial-Mesenchymal Transition (EMT) process of lung adenocarcinoma cells

**Figure 2:**
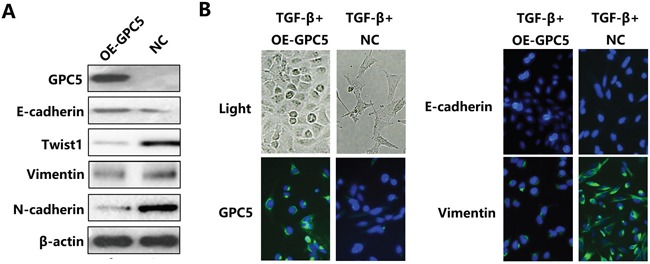
GPC5 inhibits the Epithelial-Mesenchymal Transition (EMT) process in lung adenocarcinoma cells **A.** the expression levels of GPC5, E-cadherin, Twist1, Vimentin, N-cadherin and β-actin were compared by western blotting analysis between the OE-GPC5 A549 cells and the control cells. **B.** representative images of immunofluorescence of GPC5, E-cadherin and Vimentin in TGF-β induced OE-GPC5 cells and TGF-β induced control cells (left panel and right panel, respectively). Light microscope was used to contrast (left, top).

After overexpressing GPC5, Western blot analysis indicated that the E-cadherin, the marker for epithelial cells, was up-regulated, while Vimentin, N-cadherin and Twist1, markers for interstitial cells, were all down-regulated (Figure 2A). After conducting TGF-β stimulation, negative control A549 cell lines exhibited fusiform morphology. Subsequently, the reversion of EMT process was observed in TGF-β stimulated OE-GPC5 A549 cell lines. In addition, the outcomes of immunofluorescence was consistent with western blot. The bright green immunofluorescence is observed in TGF-β induced OE-GPC5 cells of E-cadherin and OE-GPC5 cells of Vimentin (Figure 2B). In consequence, we discovered that over expressing GPC5 could reverse the EMT process in A549 lung adenocarcinoma cell lines.

### Overexpressing GPC5 inhibits the Epithelial-Mesenchymal Transition (EMT) via inactivating Wnt/β-catenin signaling pathway

**Figure 3:**
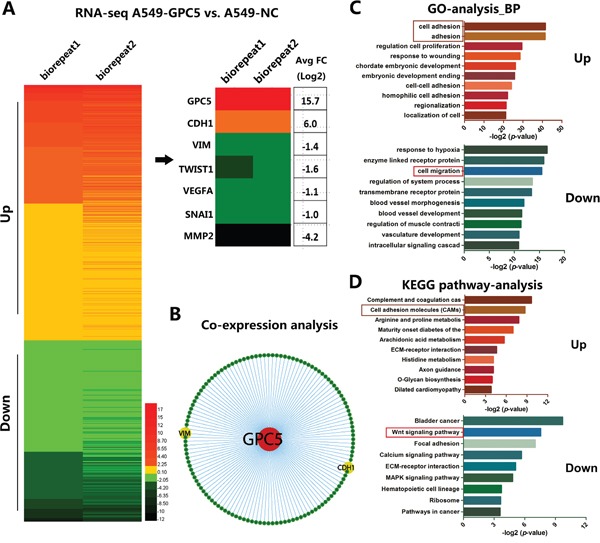
Bioinformatic analysis of different expression genes after overexpressing GPC5 in A549 cell lines **A.** Heat map diagrammed up and down regulated genes in OE-GPC5 A549 cells. **B.** E-cadherin (CDH1) and Vimentin (VIM) were obtained from Co-expression analysis. **C.** Gene Ontology (GO) analysis observed up-regulated genes are associated with cell adhesion and down-regulated genes are relevant to cell migration. **D.** KEGG pathway enrichment analysis indicated up-regulated genes are associated with cell migration molecules and down-regulated genes are relevant to Wnt signaling pathway.

We subsequently carried out transcriptome analyses (RNA-sequence) in OE-GPC5 A549 cells and control cells. The heat map of RNA-seq depicted: after overexpressing GPC5 in A549 cell lines, the expression level of 877 genes doubled the increase while 1232 genes doubled the decrease compared with the control (Figure 3A, left). The expression of CDH1 (E-cadherin) was significantly up-regulated after overexpressing GPC5 in A549 cells, while Vimentin, Twist1, Snail1, VEGFA and MMP2 were all significantly down-regulated (Figure 3A, right). In addition, co-expression analysis also indicates that GPC5 is associated with E-cadherin (CDH1) and Vimentin (VIM) significantly (Figure 3B). The outcomes therefore suggested that GPC5 might affect lung cancer cell migration via regulating the EMT process. More importantly, Gene Ontology (GO) analysis of biological process indicated up-regulated genes were associated with cell adhesion and down-regulated genes were associated with cell migration (Figure 3C). Additionally, KEGG pathway enrichment analysis suggested that up-regulated genes were associated with cell adhesion molecules and down-regulated genes were associated with Wnt signaling pathway (Figure 3D). Therefore, we supposed that GPC5 could inhibit the EMT process via regulating Wnt/β-catenin signaling pathway.

**Figure 4:**
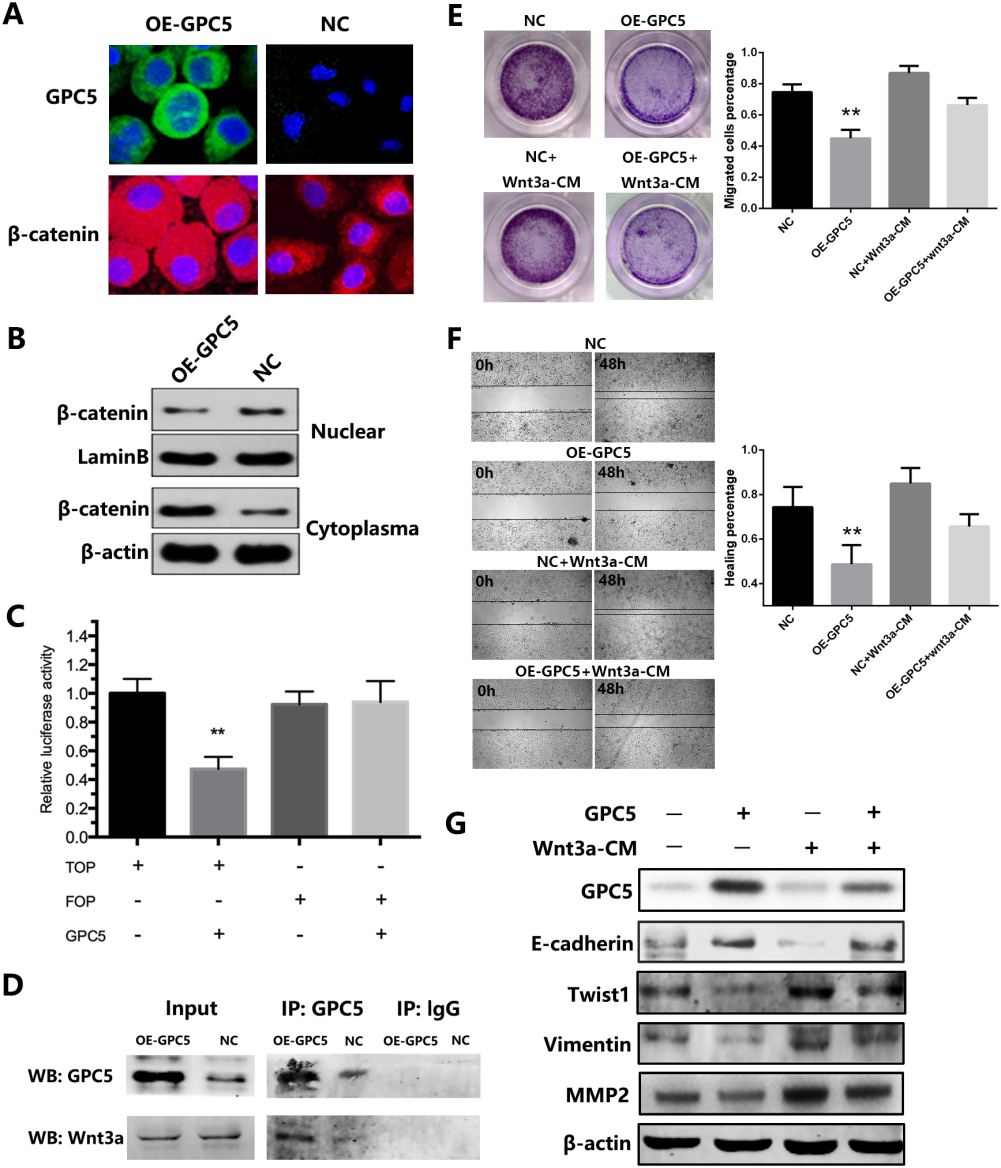
GPC5 inhibits Wnt/β-catenin signal pathway via competitively binding to Wnt3a **A.** immunofluorescence staining of GPC5 (FITC, green, magnification: ×600) and β-catenin (TRITC, red, magnification: ×600) in OE-GPC5 A549 cells (left) and in respective control cells (right). **B.** the expression levels of β-catenin in nuclear and cytoplasma were compared by western blotting analysis between the OE-GPC5 A549 cells and the control cells. LaminB and β-actin were loading control respectively. **C.** Wnt signaling in OE-GPC5 A549 cells was detected by the TOP/FOP flash Wnt reporter assay **D.** in Co-immunoprecipitation assay, GPC5 was used to pull-down Wnt3a both in OE-GPC5 A549 cells and the control. **E.** rescue of migration by adding Wnt3a in cell culture medium of OE-GPC5 A549 cells. Representative images of transwell membrane insert in 24-well plate (left). The results were plotted as the percentage of migrated cells (right). **F.** representative images of Wound healing assay by adding Wnt3a in cell culture medium of OE-GPC5 A549 cells (left). And the results were plotted as the percentage of healing (right). **G.** the expression levels of GPC5, E-cadherin, Twist1, Vimentin and MMP2 were compared by the western blotting analysis between 4 groups in this rescue experiment.

To our knowledge, the accumulation of β-catenin in cytoplasm indicates the Wnt signaling pathway is stimulated. In order to determine whether β-catenin sub-cellular location was affected by GPC5 or not, we performed the immunofluorescence and nuclear & cytoplasmic fraction of β-catenin in A549 cells. We observed that β-catenin translocated from nuclei to cytoplasm in OE-GPC5 A549 cells (Figure 4A & 4B). Subsequently, to investigate whether the transcriptional activity of downstream β-catenin/TCF complex, the TOP & FOP-Flash Wnt reporter assay was performed and we found that overexpressing GPC5 significantly inhibited Wnt signaling in A549 cell lines (Figure 4C). To further clarify the underlying mechanisms, we took co-immunoprecipitation assay. The result observed that GPC5 could physically bind with Wnt3a (Figure 4D). Finally, in order to confirm the above mechanism, we designed a rescue experiment by using Wnt3a conditioned media (Wnt3a-CM). As shown in Figure 4E & 4F, the invasion and migration abilities of OE-GPC5 A549 cells cultured in Wnt3a-CM were at partially increased as compared with OE-GPC5 A549 cells in normal media. Additionally, the protein level alternation of EMT related genes, including E-cadherin, Twist1, Vimentin, and MMP2 were all accordingly rescued in Wnt3a-CM cultured OE-GPC5 A549 cells (Figure 4G).

### GPC5 inhibits lung adenocarcinoma and Epithelial-Mesenchymal Transition (EMT) in NOD-SCID mice model

**Figure 5:**
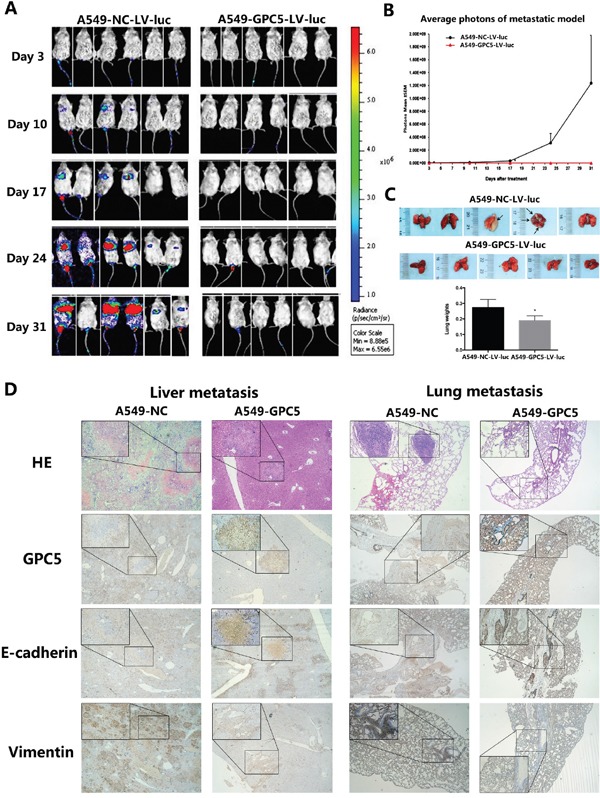
GPC5 inhibits lung adenocarcinoma and Epithelial-Mesenchymal Transition (EMT) in NOD-SCID mice model **A.** LAC metastatic model *in vivo*. 4.0×10^6^ A549-NC-L.V-luc and A549-GPC5-L.V-luc cells were injected by tail vein into SCID mice imaged for bioluminescence and analyzed by the number of photons. **B.** the change of average photon intensities was demonstrated by the line graph. **C.** representative image of the visible metastatic nodules in the mouse lungs of the A549-NC-LV-luc group and the A549-GPC5-LV-luc group (n=5, top). Average weight of the mouse lungs in the subject and the control groups were presented as the mean ± SD (bottom). **D.** representative HE and immunofluorescence staining images of the visible metastatic nodules from the mouse liver and lung sections of the A549-NC group and the A549-GPC5 group.

In LAC metastatic model, we subsequently injected A549-NC-LV-luc cells and the A549-GPC5-LV-luc cells into the tail vein of the NOD-SCID mice. All SCID mice presented bioluminescent signal at 3^rd^ day, with a constant increase until the end at 31^st^ day (Figure 5A). The line graph demonstrated that migration and proliferation of A549-GPC5-L.V-luc group was significantly weaker than the control, especially on 24^th^ and 31^st^ day (*p* < 0.05) (Figure 5B). Visual and microscopic evaluation of the metastatic growth in the lungs of the SCID mice showed lung metastasis numbers were significantly reduced compared to the control (Figure 5C, top). In addition, the lung tissues of the A549-GPC5-L.V-luc group are significantly lighter than A549-NC-L.V-luc group (*p* < 0.05). The outcomes of HE and immunofluorescence staining observed that the epithelial mark E-cadherin was significantly up-regulated and the mesenchymal mark Vimentin was simultaneous significantly down-regulated, both in the liver and lung metastatic nodules (Figure 5D).

### GPC5 expression was significantly correlated with the expression of E-cadherin, Twist1, and MMP2

**Figure 6:**
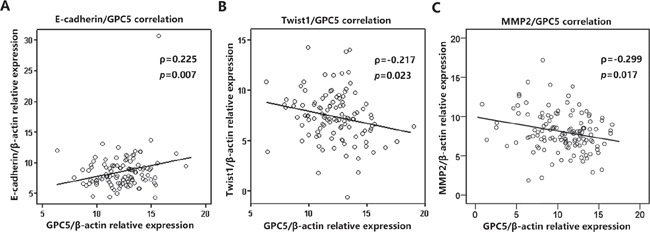
GPC5 expression was significantly correlated with the expression of E-cadherin, Twist1, and MMP2 in NSCLC The expression of E-cadherin, Twist1 and MMP2 were all detected in 198 paired NSCLC tissue samples by real-time RT-PCR analysis. Pearson correlation analysis indicated that E-cadherin, Twist1 and MMP2 are all highly correlated with GPC5 (**A., B., C.,** respectively).

To verify the findings in LAC patients, we detected the expression of E-cadherin, Twist1 and MMP2 in 134 paired LAC tissue samples by real-time RT-PCR analysis. Consequently, Pearson correlation analysis discovered that GPC5 and E-cadherin is significant positively correlation (ρ=0.225, *p*=0.007, Figure 6A), while Twist1 and MMP2 were all negatively correlated with GPC5 (ρ= −0.217, *p*=0.023 and ρ=−0.299, *p*=0.017 respectively, Figure 6B & 6C).

## DISCUSSION

In the present study, by analyzing TCGA data, we confirmed that GPC5 was generally downregulated in LAC tissues and much more significantly down-regulated in stage N1-2 groups than N0 group. By performing invasion and migration assay *in vitro* and establishing LAC metastatic NOD-SCID mice model *in vivo*, it was indicated that the inhibition of EMT process contributes to the LAC metastasis suppression of GPC5. More importantly, we demonstrated that GPC5 could competitively bind to Wnt3a so as to inactivate the canonical Wnt/β-catenin pathway, which contributes the subsequent inhibition of EMT process and LAC metastasis.

GPC5 is one of the six members of the glypican family which regulate the interaction between growth factor and receptor [11]. So far, the role of GPC5 related to cancer is far from clear. Notably, the role of GPC5 varied across different cancer types. For example, GPC5 could stimulate cell proliferation in rhabdomyosarcoma, which suggested that GPC5 acted as an oncogene [6]. Zhang et al. [12] reported that GPC5 was significantly up-regulated in lung metastasis of salivary adenoid cystic carcinoma (SACC), which suggested GPC5 might promote lung metastasis in SACC. However, in breast cancer, GPC5 expression was further decreased in malignant breast tumors in comparison to begin tumors, suggested that GPC5 played a role of tumor suppressor [4]. Because of a genome-wide association study (GWAS) in never-smokers' lung cancer, GPC5 was firstly linked with lung cancer [7]. Subsequently, we firstly demonstrated that GPC5 was generally downregulated in NSCLC, negatively correlated with lymph node metastasis and patients' prognosis, as well as suppressing the NSCLC invasion and migration *in vitro* [9]. In the present study, the third-party data from TCGA once again confirmed our previous findings and the metastasis suppressor property of GPC5 was further verified by LAC metastasis SCID mice model *in vivo*. In addition, a lasted study reported by Yuan et al. [13] also achieved similar results, and all these evidences support our finding that GPC5 is a lung cancer metastasis suppressor.

Since the biological behavior of GPC5 appears totally different in various cancers, we have reason to believe that there might be a unique molecular mechanism utilized by GPC5 in lung cancer. GPC5 belongs to the HSPGs family, a group of membrane-attached proteins with the GPI anchor, which indicates that they might sever as a “hub” attracting many proteins, such as cytokines, chemokines, and growth factors [8]. In rhabdomyosarcoma, previous studies demonstrated that GPC5 could bind with Hedgehog (Hh) and then activate Hh signaling pathway [6]. However, in our study, after overexpressing GPC5 in A549 cells, RNA-seq data indicated that EMT related genes were co-expressed with GPC5 and Wnt signaling pathway was significantly enriched, whereas Hh pathway relative genes were not altered by GPC5. The canonical Wnt/β-catenin pathway is usually activated by secreting Wnt family proteins which is similar to Hh signaling pathway [14]. And also, GPC3, another GPC member, was previously demonstrated to inhibit canonical Wnt pathway and suppress breast cancer metastasis [15]. We therefore hypothesized that GPC5 could also inactivate canonical Wnt pathway by competitively binding to Wnt family proteins. Finally, we found that GPC5 could only bind to Wnt3a and subsequently inhibit Wnt signaling pathway. Recent study reported by Yuan et al. [13] also supported our findings.

**Figure 7:**
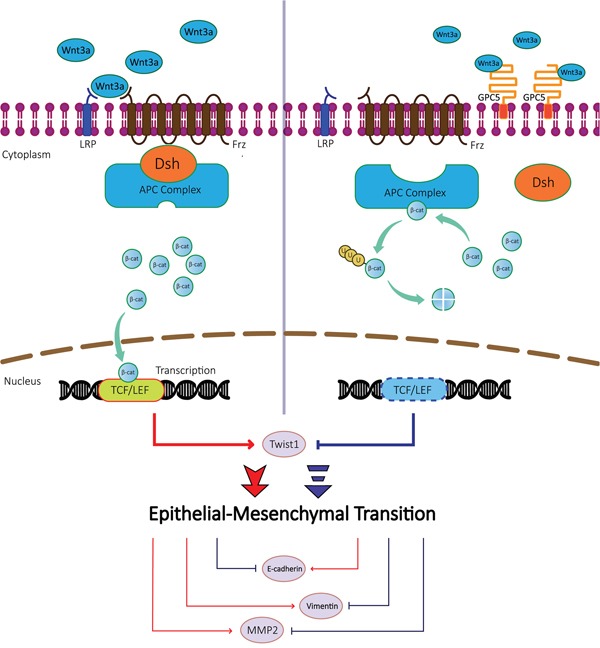
A schematic diagram of GPC5 based signaling circuit in lung adenocarcinoma The diagrammatic process of regulating the Wnt/β-catenin signaling pathway and downstream effector proteins. In lung adenocarcinoma cells, GPC5 inhibits Epithelial-Mesenchymal Transition (EMT) by competitively binding to Wnt3a.

To our knowledge, the EMT process plays a significant role in the tumor metastasis [16, 17]. According to our previous and current studies, the overexpression of GPC5 was found significantly correlated to the EMT. EMT associated genes, such as E-cadherin, Twist1 and MMP2, could be regulated by the canonical Wnt/β-catenin signaling pathway. E-cadherin is a molecular marker of the EMT, and the inhibition of the canonical Wnt/β-catenin pathway could activate the expression of E-cadherin [18, 19]. In our study, E-cadherin was indicated positively correlated to the GPC5 growth. Twist1 could induce the EMT process in tumors [20, 21]. In our study, Twist1 was discovered negatively correlated with the increased GPC5 expression and could be regulated by the canonical Wnt/β-catenin signaling pathway. MMP2, the gene down-regulated most obviously in the heat map, was demonstrated activating TGF-β to promote the EMT [22, 23]. Additionally, MMP2 was regulated by Wnt/β-catenin pathway and significant negatively correlated with the increase expression of GPC5 [19, 24]. Thus, we propose that the GPC5 protein could bind to Wnt3a at the cell surface and subsequently inhibit the canonical Wnt/β-catenin signaling pathway. When the expression of GPC5 is inhibited, Wnt3a could bind with Frizzled to activate Wnt/β-catenin signaling pathway, which will induce the process of EMT (Figure 7).

In conclusion, the present study suggests that GPC5 is able to suppress the EMT and metastasis of LAC cells by inhibiting the canonical Wnt/β-catenin signaling pathway. The low expression of GPC5 could predict lymph node metastasis and poor survival of lung adenocarcinoma. All the current findings highlight the significance of GPC5 in tumor progression and suggest GPC5 might be an important novel biomarker in lung adenocarcinoma.

## MATERIALS AND METHODS

### Patient samples

A total of 134 freshly resected LAC specimens and adjacent normal lung tissues from 198 NSCLC tissue samples were collected from the Department of Thoracic Surgery at the Nanjing Medical University Affiliated Cancer Hospital [9]. The specimens were snap-frozen in liquid nitrogen. The patient samples were obtained following informed consent according to an established protocol approved by the Ethics Committee of Nanjing Medical University. The descriptive clinical characteristics of the samples was summarized in Supplementary Table S1.

### Data sources and bioinformatics

Level 3 TCGA data: TCGA_LUNG_exp_HiSeqV2 were downloaded at the website of the UCSC cancer browser (http://genome-cancer.ucsc.edu). All mRNA expression values were normalized, and values for GPC5 expression were obtained from the “genomicMatrix” file (using Editplus^®^ software). Fisher's t-test and variance analysis were used to compare the two groups.

### RNA extraction and quantitative real-time RT-PCR

The total RNA was extracted from tissues or cultured cells with Trizol reagent (Life Technologies, Scotland, UK) according to the manufacturer's protocol. A 1.5-μg total RNA was reverse transcribed in a final volume of 20 μl using random primers under standard conditions using the PrimeScript RT Master Mix (Takara, Cat. # RR036A).

Quantitative reverse transcription RT-PCR (qRT-PCR) was carried out as described previously [25]. The CT-value for each sample was calculated with the ΔΔCT-method, and the results were expressed as 2^−ΔΔCT^ to analyze the fold change (tumor vs. normal): ΔΔCT= (CT_target gene_-CT_actin_) _normal_-(CT_target gene_-CT_actin_) _tumor_. The sequences of PCR primers were according to the previous study [9].

### Cell culture

The human LAC cell lines, A549 from a smoker with LAC, was obtained from American Type Culture Collection (ATCC, USA). The two LAC cell lines were cultured in RPMI-1640 (HyClone) supplemented with 10% fetal bovine serum (FBS, Invitrogen). Cell cultures were maintained under an atmosphere containing 5% CO_2_ in 37°C (Thermo Scientific Forma).

### Lentivirus production and overexpression of GPC5 in cell lines

Human GPC5 cDNA was subcloned in the plasmid pLenti6/V5 (Invitrogen). A549 cell line was transfected with GPC5 or empty control recombinant lentivirus labeled with luciferase (L.V-GPC5 or L.V-Empty, respectively) using the Lipofectamine™ 2000 transfection reagent according to the manufacturer's instructions. Stable GPC5-overexpressing LAC cell lines were successfully constructed.

### Establishment of LAC metastatic model *in vivo*

All severe combined immune deficiency (SCID) mice were housed and handled under sterile conditions in facilities accredited by the American Association for the Accreditation of Laboratory Animal Care (AAALAC). We choose A549 cell line with stronger metastatic ability to establish the metastatic model. A total of 12 female SCID mice, 6-8 weeks old, were divided into two groups. Each cell line as a group contained 6 mice. A549-NC-L.V-luc and A549-GPC5-L.V-luc cell lines were cultured to the logarithmic growth phase. Mestastic models were established by tail vein injection of 4.0×10^6^ cells. When the mice were sacrificed after 40 days, lungs were isolated and weighed. The bioluminescence imaging was used to monitor tumor metastasis on 3^rd^, 10^th^, 17^th^, 24^th^, 31^st^ day after inoculating cell lines.

### Establishment of LAC orthotopic model *in vivo*

We choose A549 cell line to establish the orthotopic model. A total of 12 female SCID mice, 6-8 weeks old, were divided into two groups. Each cell line as a group contained 6 mice. Orthotopic models were also established in mice by injection of 2.0×10^6^ A549-NC-L.V-luc or A549-GPC5-L.V-luc cells in lungs directly. The bioluminescence imaging was also conducted every week. The body weights of mice were measured twice a week. The survival curves were drawn at the end of experiment.

### Bioluminescence imaging

As the reference described [26], mice received 150 mg/kg D-Luciferin (Caliper Life Science Part Number #119222) intraperitoneally, were anesthetized with 2.5-3.5% isoflurane, and imaged after 15 min, which is the peak of maximum bioluminescence, with an IVIS Lumina XR imaging system (Caliper, USA). The whole body was imaged in a supine gesture. Bioluminescence intensity is expressed as photons per second (p/s). Then the mice were returned to their cages where they awake quickly.

### Microarray analysis

Microarray flip dye experiments on cancer cell lines were performed with Human OneArrayTM Whole Genome Microarray v 6.1 (Phalanx Biotech). Cell lines were cultured for 24 hours. RNA was extracted were sent to Phalanx Biotech Group for microarray analysis. All the genes identified in the microarray experiment were ontologically classified using the PAnTher classification system and grouped according to cellular pathways and biological processes.

### Immunofluorescence and western blot

As previous described, the expression alteration of β-catenin and GPC5 was both detected by Immunofluorescence [12] and western blot [6]. The β-catenin was labelled with fluorescein isothiocyanate (FITC) and the GPC5 was labeled with tetramethyl rhodamine isothiocyanate (TRITC). WNT/β-catenin signaling pathway-related proteins (wnt3a, wnt5a/b, cyclinD1, MMP2, MMP7, MMP9, E-cadherin, β-catenin) and internal control proteins (LaminB, β-actin, Gapdh) antibodies were purchased from Cell Signaling Technology, Inc. (Beverly, MA, USA). Protein density on scanned western blots was analyzed using Image J 1.44 software, and we performed semi-quantitative analysis for the results of the western blot analysis.

### Nuclear and cytoplasmic fractions

The subcellular localization of β-catenin was detected by PARIS ™ Kit (Ambion, Life Technologies, USA) according to the manufacturer's protocol. About 2*106 cells were collected and washed in cold PBS. After resuspending the cells in 500 μl ice-cold Cell Fractionation Buffer, the cytoplasmic fraction was aspirated away from the nuclear pellet which was lysed in Cell Disruption Buffer subsequently.

### Wnt reporter assays

Luciferase assays for reporters were carried out using the Dual-Luciferase Reporter Assay System (Promega, Madison, WI) as reported previously. A549 cells were plated at a concentration of 5000 cells/well on 96 well plates. Lipofectamine 2000 (Invitrogen, Carlsbad, CA) was used to mediate co-transfection of 0.2 mg TOP-flash or FOP-flash expression plasmids (Millipore, Temecula, CA, USA). The Renilla luciferase reporter vector pRL-TK (0.02 μg) (Promega, Madison, WI) was simultaneously transfected as the control. TCF-mediated transcriptional activity was determined by the ratio of the TOP/FOP ratio, each normalized to the luciferase activities of the pRL-TK reporter [27].

### Immunoprecipitation

A549 cells were used for each experiment. Cells were suspended in 500 μl lysis buffer (20 mM Tris, 2.5 mM NaCl, 1% Triton X-100, pH 7.4) supplemented with protease inhibitor mix, disrupted for 20′ at 4°C on a rotary shaker and centrifuged at 13000 × g. The pellet was discarded and 4 μl of anti-Wnt3a antibody were added except to the control samples. The mixture was incubated overnight at (ca. 15 h) 4°C on a rotary shaker. A Protein-G agarose resin was then added and samples were incubated for further 3 h at 4°C. After that, samples were centrifuged, the supernatant was removed and the protein-G agarose resin was washed 5 times with washing buffer (50 mM Tris, 150 mM NaCl, 0.1% Triton X-100, pH 7.4). Lämmli buffer was added directly to the protein-G agarose, heated at 95°C, probes were run on a SDS–PAGE gel and finally detected by staining.

### Transwell invasion assay

Cell invasion assays were performed using 24-well transwells (8-mm pore size, Corning Life Sciences) coated with 1mg/mL Matrigel (BD Science). The cells were seeded in the upper chamber of the wells in 100 μl FBS-free medium, and the lower chambers were filled with 500μ1 20% FBS medium. Following incubation for 24h, the cells on the filter surface were fixed with 4% formaldehyde, stained with 0.1% crystal violet, and photographed with a phase-contrast inverted microscope. The cells from at least five random microscopic fields (100x) were counted.

### Monolayer wound healing assay

Migration ability was measured using a wound-healing assay. The cells were grown in 10% FBS medium on 60-mm plates. Once the cells reached 60% density, the monolayer was scratched and then incubated in fresh medium for 24 h. The width of the wound was measured after 24 h. Three different locations were visualized and photographed with a phase-contrast inverted microscope (100x objective, Leica, Solms, Germany).

### Statistical analysis

Results are expressed as mean ± standard deviation (SD). Comparisons of the quantitative data were performed using a two-tailed student's t test. Correlation analysis was performed using the Spearman and Pearson test. A *p* value less than 0.05 was considered statistically significant. The data were analyzed using SPSS 18.0 and GraphPad Prism 6.0 software.

## SUPPLEMENTARY TABLES


