# Supplementary material for: Glypican-5 suppresses Epithelial-Mesenchymal Transition of the lung adenocarcinoma by competitively binding to Wnt3a
Source: Oncotarget. 2016 Oct 27;7(48):79736–46. doi: 10.18632/oncotarget.12945 (PMC5346747; doi:10.18632/oncotarget.12945)
Supplement: Supplementary file 1 [file oncotarget-07-79736-s001.pdf]

Glypican-5 suppresses Epithelial-Mesenchymal Transition of the lung adenocarcinoma by competitively binding to Wnt3a

SUPPLEMENTARY TABLES

Supplementary Table S1: The correlation between decreased expression of GPC5 in the LAC (tumor vs. normal) and clinicopathologic factors

Variables		Number of cases	Decreased expression of GPC5 mRNA in the tumor (vs. normal)	*P* value
**Sex**	Male	67(50.0%)	-10.56±4.75	0.147
	Female	67(50.0%)	- 6.95±2.74	
**Age**	<60 years	78(58.2%)	-11.25±4.89	0.781
	≥60 years	56(41.8%)	-24.87±3.12	
**Smoking status**	Smoker	70(52.2%)	-10.37±4.31	0.391
	Non-smoker	64(47.8%)	-7.13±2.11	
**T stage**	T1	70(52.2%)	-6.76±3.82	0.490
	T2	58(43.3%)	-9.87±5.01	
	T3	6 (4.5%)	-21.25±10.43	
**N stage**	N0	84(62.7%)	-6.26±4.20	0.021*
	N1-2	50(37.3%)	-12.94±7.82	
**pTNM stage**	I	73(54.5%)	-6.12±4.63	0.015*
	II	15(11.2%)	-9.56±3.35	
	III-IV	46(34.3%)	-12.67±2.64	
**Differentiation**	High	6(4.5%)	-0.71±0.20	0.001*
	High-medium	11(8.2%)	-1.11±0.44	
	Medium	50(37.3%)	-6.58±1.83	
	Medium-low	41(30.6%)	-7.85±2.42	
	Low	26(19.4%)	-19.01±2.66	

Supplementary Table S2: Spearman correlation analysis of relative GPC5 expression and clinicopathologic factors

Variables	Spearman correlation test (*p* value)<br>Fold change of GPC5 expression in tumor (vs. normal)
**T stage**	0.166(0.056)
**N stage**	0.221(0.010*)
**pTNM stage**	0.242(0.005*)
**Differentiation**	0.311(<0.001*)
**Smoking**	-0.189(0.058)
